# A single-nucleotide polymorphism of miR-146a and psoriasis: an association and functional study

**DOI:** 10.1111/jcmm.12359

**Published:** 2014-09-11

**Authors:** Weigang Zhang, Xiuli Yi, Sen Guo, Qiong Shi, Chao Wei, Xia Li, Lin Gao, Gang Wang, Tianwen Gao, Lei Wang, Chunying Li

**Affiliations:** Department of Dermatology, Xijing Hospital, Fourth Military Medical UniversityXi’an, Shaanxi, China

**Keywords:** psoriasis, microRNA, SNP, miR-146a, *EGFR*

## Abstract

Epidermal growth factor receptor (*EGFR*), which is overexpressed in psoriatic lesions, has been proven to contribute to the hyperproliferation of keratinocytes in psoriasis. Single nucleotide polymorphisms (SNPs) involved in miRNAs that can regulate the expression of *EGFR* could potentially influence the development of psoriasis. The present study investigated the association between a functional SNP of rs2910164 in miR-146a and the risk of psoriasis in the Chinese Han population. A total of 521 Han Chinese patients with psoriasis and 582 healthy controls were recruited in this study. The miR-146a rs2910164 SNP was genotyped by polymerase chain reaction-restriction fragment length polymorphism. Overall, a significantly increased risk of psoriasis was associated with the rs2910164 miR-146a CG and GG genotypes (adjusted OR, 1.38; 95% CI, 1.06–1.80). Furthermore, the rs2910164G allele in miR-146a attenuated its inhibitory regulation on the expression of *EGFR* as well as the proliferation of human keratinocytes, and lowered the level of miR-146a in the psoriatic lesions. These findings indicate that the rs2910164G allele in miR-146a weakens its suppression on the proliferation of keratinocytes probably through the decreased inhibition of the target gene, *EGFR*, which may account for the increased risk of psoriasis in this study population.

## Introduction

Psoriasis is a common inflammatory skin disease, with an overall prevalence of 2–3% of the world’s population 1. Clinically characterized by raised, well-demarcated, erythematous oval plaques with adherent silvery scales 2, this cutaneous disease can impose a substantially negative impact on the patients’ quality of life. Although the pathogenesis of psoriasis is still less obvious, various theories, including genetic predisposition, autoimmunity, infectious background and metabolic disorders, have been considered to contribute to the development of psoriasis 3. The main pathological feature of psoriasis is the hyperproliferation of keratinocytes in the epidermis 4, which has been identified as a key event in the development of the disease 5. Some recent studies have suggested that epidermal growth factor receptor (*EGFR*), a cell surface protein that binds to epidermal growth factor, is overexpressed in psoriatic lesions 6 and contributes a lot to the hyperproliferation of keratinocytes in psoriasis 7,8. Moreover, a drug called SU5271 that inhibits the activity of *EGFR* has been verified to suppress the proliferation of keratinocytes in an initial clinical trial 9, which demonstrates that *EGFR* can act as a therapy target for psoriasis.

MicroRNAs (miRNAs) are endogenous non-coding small RNAs that can regulate gene expression at the post-transcriptional level. They function by binding to complementary sites in the 3′ untranslated region (UTR) of target mRNA, thus impeding translation of mRNAs or, less frequently, leading to degradation of target mRNAs 10. As important gene regulators, miRNAs have been verified to take part in various physiological processes of humans 11, and their aberrant expression is involved in the pathogenesis of many diseases. Moreover, recent evidence has shown that single nucleotide polymorphisms (SNPs) in miRNAs could influence their expression and function 12,13, thus affecting the phenotypes and development of many diseases 14–16.

**Figure 1:**
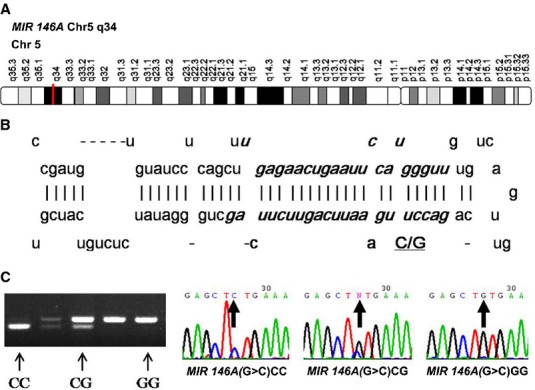
Location and detection of the *MIR146A* gene and rs2910164. (**A**) Location of the *MIR146A* gene. (**B**) Location of the rs2910164 SNP. (**C**) Genotypes of rs2910164 in the *MIR146A* gene and sequence analyses of the *MIR146A* polymerase chain reaction products.

The *MIR146*A gene maps to chromosome 5q34, encoding miR-146a that has been widely studied in various diseases (Fig. 1A). A single-nucleotide of polymorphism called rs2910164 was located within the miR-146a precursor, which stands for a change from G allele to C allele in its seed sequence (Fig. 1B). This SNP has been shown to affect the level of mature miR-146a 17,18 and associated to the risk of various cancers and inflammatory diseases 19–22. However, little is known regarding the contribution of rs2910164 to the development of psoriasis. In 2011, Kogo *et al*. reported that miR-146a can target the *EGFR* mRNA 23, thus down-regulating the protein level of the gene. Given the regulatory relation between miR-146a and *EGFR* as well as the important role of *EGFR* in the development of psoriasis, we have suggested that rs2910164 in miR-146a could potentially affect the miR-146a-mediated regulation on *EGFR*, thus leading to individual susceptibility to psoriasis.

To test our hypothesis, we first performed a hospital-based case–control study to evaluate the association between the rs2910164G > C SNP within pre-miR-146a and the susceptibility to psoriasis. Moreover, we compared the lesional level of miR-146a in psoriasis patients and healthy controls with different genotypes to determine whether the rs2910164G > C SNP within pre-miR-146a seed can affect mature miR-146a levels *in vivo*. In addition, several *in vitro* experiments referring to the function of rs2910164 SNP in keratinocytes and *EGFR* regulation were also performed to elucidate its functional relevance with the development of psoriasis.

## Materials and methods

### Study participants

DNA samples from 521 psoriasis cases and 582 healthy controls were collected from Xijing Hospital, Fourth Military Medical University. Only Han Chinese (more than 90% of Chinese populations) patients were included in this study to avoid variations in genotype frequencies among different ethnic groups. The controls were persons who presented for health examinations and were frequency-matched to the cases by age (±5 years), sex and ethnicity. A standard questionnaire was used to obtain demographic and characteristic information (*e.g*., age, sex, onset age, disease course and family history). The Psoriasis Area and Severity Index (PASI) score of each psoriasis patient was evaluated by dermatologists. Informed consent was obtained from all the patients and healthy controls. The research protocol was designed and performed according to the principles of the Helsinki Declaration and was approved by the ethics review board of the Fourth Military Medical University.

### Genotyping

Genomic DNA was extracted from the peripheral blood by using a DNA isolation kit (Tiangen, Beijing, China). The rs2910164 SNP in miR-146a was detected by using polymerase chain reaction-restriction fragment length polymorphism (PCR-RFLP) analysis. PCR amplifications were generated by using the following primers: forward 5′-CATGGGTTGTGTCAGTGTCAGAGCT-3′ and reverse 5′-TGCCTTCTGTCTCCAGTCTTCCAA-3′ (product of 147 bp). PCR was performed with 50 ng of DNA as a template under the following conditions: 95°C for 5 min.; 32 cycles of 95°C for 30 sec., annealing temperature of 56°C for 30 sec. and 72°C for 40 sec.; and final extension step at 72°C for 10 min. After affinity membrane purification, the PCR products were subjected to cycle sequencing with the respective forward and reverse primers. The rs2910164 SNP was delineated by using the SacI restriction enzyme (New England Biolabs, Ipswich, MA, USA), which cuts only the C allele, resulting in 125- and 22-bp fragments (Fig. 1C). More than 10% of the samples were randomly selected and genotyped again by the same method to test the discrepancy rate, and the results were 100% concordant.

### Collection of Skin Specimens and RNA extraction

Lesional specimens were redundant tissues from 32 psoriasis patients who accepted biopsy. 15 patients also agreed to provide their non-lesional skin samples. Healthy skin specimens were collected from 17 patients who accepted cosmetic surgery in Xijing Hospital. All the donors provided written informed consent. All the specimens were stored in RNAlater (Ambion, Austin, Texas), frozen in liquid nitrogen, and kept at−80°C until RNA extraction. Total RNA was extracted from psoriasis skin specimens as well as cultured cells by using Trizol reagent (Invitrogen, Carlsbad, CA, USA) according to the manufacturer’s protocol.

### Quantitative real-time polymerase chain reaction (qRT-PCR)

cDNA was synthesized from miRNA by using miRNA cDNA First Strand Synthesis or from mRNA by using PrimeScript RT reagent Kit (Takara, Ohtsu, Japan). Quantitative realtime PCR (qRT-PCR) for pre-miR-146a was performed with SYBR Premix Ex Taq II (TaKaRa) with a BIO-RAD Multicolour Real-time PCR Detection System (iQTM5). The primers we used are listed here: pre-miR-146a (forward 5′-TGAGAACTGAATTCCATGGGTT-3′ and reverse 5′-CTGAAGAACTGAATTTCAGAGG-3′), β-actin (forward 5′-AGAAAATCTGGCACCACACC-3′and reverse 5′-AGAGGCGTACAGGGATAGCA-3′). The cycling conditions are as follows: 95°C for 2 min., followed by 45 cycles of denaturation at 95°C for 5 sec., annealing at 57°C for 10 sec. and extension at 72°C for 15 sec. QRT-PCR for miR-146a was performed with miRNA SYBR qRT-PCR Kit (Qiagen, Valencia, CA, USA) with iQTM5. The primers of RUNU6, and hsa-miR-146a were designed and synthesized by Tiangen Company based on miRBase (http://www.mirbase.org). DNA was amplified for 40 cycles of denaturation for 20 sec. at 95°C and annealing for 60 sec. at 60°C. All reactions were run in triplicates for at least three independent experiments. Relative quantification was performed according to the ΔΔCT method, and results were expressed in the linear form by using the formula 2^−ΔΔCT^ 24.

### Cell culture

HaCaT cells were cultured in DMEM (Gibco-Invitrogen, Carlsbad, CA, USA) with 10% foetal bovine serum (Gibco-Invitrogen) under a humidified atmosphere containing 5% CO_2_ at 37°C. Normal human keratinocytes were extracted from plastic surgery skin obtained from healthy individuals. Second- or third-passage keratinocytes were used in all experiments, with cells cultured in the serum-free medium, keratinocyte growth medium (Gibco-Invitrogen). Each experiment was repeatedly performed in normal human keratinocytes at least from three different sources.

### Construction of the plasmids

To express the different precursors of miR-146a, the pri-miRNA sequence was amplified by PCR (oligonucleotide primer sequences: for Pre-mir-146a C allele, forward 5′-GGCAACAAGAAACTGcCTGAGTTACATCAGTC-3′ and reverse 5′-GACTGATGTAACTCAGGCAGTTTCTTGTTGCC-3′; for Pre-mir-146a G allele, forward 5′-CCCAAGCTTGTCTACTCTCTAGTCCTTAGGGAGGTTG-3′ and reverse 5′-CCGGAATTCCACTCACAGCTTGTCCTCCTTGG-3′) and was cloned into pEX-5 plasmids with HindIII and EcoRI digestion (Invitrogen). Human *EGFR* plasmid using pCMV6-ENTRY vector was a commercial product from Origene Company (Rockville, MD, USA). All the constructs used in the study were restriction mapped and sequenced to confirm their authenticity.

### Transient transfection

Transfections of the plasmids were performed with HaCaT cells and normal human keratinocytes by using Lipofectamine 2000 (Invitrogen) according to the manufacturer’s recommendations. All the transfections were carried out in triplicate. Null vector transfection was used as the control.

### Cell growth assay

The effect of the SNP in miR-146a on the growth of keratinocytes was determined by using the CCK-8 assay with some modifications. Briefly, HaCaT cells or normal human keratinocytes were plated at a density of 4 × 10^3^ cells/well into 96-well plates and incubated at 37°C in 5% CO_2_ for 24 hrs. The cells were then transfected with miR-146a expression plasmids (C or G allele) or control plasmids pCDNA3.1. Cells prepared for protector experiment were also transfected by the *EGFR* expression plasmids at the same time. At 48 hrs after transfection, the plates were incubated at 37°C and optical density was read 2 hrs later at a wavelength of 450 nm by using a Model 550 microplate reader (Biohit BP-800; Biohit Plc, Helsinki, Finland). The test was performed in triplicate.

### Western blotting assay

After transfection for 48 hrs, the cells were first lysed with RIPA lysis buffer (Beyotime, Shanghai, China) containing a cocktail of protease inhibitors and phosphatase inhibitors for 30 min. on ice and were then centrifuged at 10,303 g for 20 min. The total protein concentration was measured by using a Bradford assay. Equal amounts of the protein samples were separated by sodium dodecyl sulphate-polyacrylamide gel electrophoresis and blotted onto a nitrocellulose membrane (Millipore, Bedford, MA, USA). The blot was blocked for 1 hr and then incubated overnight with a primary antibody against *EGFR* (Abcam, Cambridge, UK) and β-actin (Santa Cruz, CA, USA). After extensive rinsing, the blot was incubated with HRP-conjugated secondary antibodies (Zhongshan Biotechnology, Beijing, China) for 2 hrs at room temperature (RT). The immunoreactive bands were detected with an enhanced chemiluminescence reagent (Millipore, Billerica, MA, USA).

### Statistical analysis

We used the chi-squared test to evaluate the differences in frequency distributions of selected demographic variables, including each allele and genotype of the mir-146a polymorphisms. Unconditional univariate and multivariate logistic regression analyses were performed to obtain the crude and adjusted odds ratios (ORs) for the risk of psoriasis and their 95% confidence intervals (CIs). Multivariate adjustments were made for age and sex. The genotype data were also further stratified by subgroups of sex, onset age, course, PASI and family history. *P*-values of <0.05 were considered statistically significant. All tests were two-sided for statistical significance and carried out by using SAS software (version 9.1; SAS Institute Inc., Cary, NC, USA). Each functional experiment was performed at least three times, and the statistical analysis was performed with GraphPad Prism 4 software (GraphPad, San Diego, CA, USA) with the unpaired *t*-test. The means ± SD of the calculated values were used for documentation.

## Results

### Characteristics of study participants

This analysis included 521 Han Chinese patients with psoriasis and 582 age- and sex-matched controls. All the participants were examined and diagnosed by dermatologists. The frequency distributions of the selected characteristics of the cases and controls are shown in Table 1. The mean age was 32.1 ± 13.6 years old for the cases and 31.0 ± 11.2 years old for the controls (*P* = 0.137), while the proportion of men was 54.7% for the cases and 56.0% for the controls (*P* = 0.662; Table 1). The patients were considered to have a family history of psoriasis if they had one or more first- to third-degree relatives afflicted with this condition. Patients whose age of onset was less than 40 years were considered to have early-onset psoriasis, and duration less than 12 months was regarded as short course. In total, the PASI scores of 350 patients were less than 20, while the other 171 ones were higher than 20.

**Table 1:** Clinical characteristics of the psoriasis patients (cases) and controls

	Cases, n (%) (*n* = 521)	Controls, n (%) (*n* = 582)
Age (years, <20/>20)	119 (22.8)/402 (77.2)	128 (22.0)/454 (78.0)
Number of female/male individuals	236 (45.3)/285 (54.7)	256 (44.0)/326 (56.0)
Early onset/late onset	474 (91.0)/47 (9.0)	
Short course/long course	158 (30.3)/363 (69.7)	
With/without family history	125 (24.0)/396 (76.0)	
PASI (<20/>20)	350 (67.2)/171(32.8)	

Two-sided chi-squared test for the frequency distribution of selected variables between the cases and controls.

### Genotype distribution of the rs2910164 SNP in miR-146a in cases and controls

The genotype distribution of rs2910164 SNP in miR-146a in psoriasis patients and the controls as well as their associations with risk of psoriasis are shown in Table 2. The rs2910164 genotype in miR-146a frequencies observed among the controls were in agreement with the Hardy–Weinberg equilibrium (*P* = 0.184 for rs2910164 in miR-146a). The frequencies of the CC, CG and GG genotypes for the miR-146a polymorphism were 25.3%, 53.0% and 21.7%, respectively, among the cases, and 31.8%, 51.5% and 16.7%, respectively, among the controls and the difference was statistically significant (*P* = 0.021). When we used the CC genotype as the reference, a statistically significant increased risk of psoriasis was associated with the CG (adjusted OR, 1.28; 95% CI, 0.97–1.68) and GG (adjusted OR, 1.65; 95% CI, 1.16–2.35) genotypes. The frequency of the combined miR-146a variant genotype (CG + GG) was significantly higher among cases (74.7%) than among controls (68.2%; *P* = 0.018) and was associated with a significant increase in risk of psoriasis (adjusted OR, 1.38; 95% CI, 1.06–1.80). Consistent with the genotype distributions of rs2910164 SNP in miR-146a, the frequency of the miR-146a-G allele was significantly higher among patients with psoriasis than among the controls (0.482 and 0.424, respectively, *P* = 0.007).

**Table 2:** Genotype and allele frequencies of the rs2910164 in miR-146a polymorphism in psoriasis cases and controls and their associations with risk of psoriasis

Genotypes	Cases (*n* = 521)	Controls (*n* = 582)*	*P*†	Adjusted OR (95% CI)‡
	*n* (%)	*n* (%)		
miR-146a			0.021	
CC	132 (25.3)	185 (31.8)		1.00 (reference)
CG	276 (53.0)	300 (51.5)	0.072	1.28(0.97–1.68)
GG	113 (21.7)	97 (16.7)	0.006	1.65(1.16–2.35)
CG + GG	389 (74.7)	397 (68.2)	0.018	1.38(1.06–1.80)
G allele	0.482	0.424	0.007	

* The observed genotype frequencies among the control participants were in agreement with the Hardy–Weinberg equilibrium (χ^2^ = 1.764, *P* = 0.184 for rs2910164 in miR-146a).

† Two-sided chi-squared test for distributions of genotype or allele frequencies between the cases and controls.

‡ Odds ratios (ORs) were obtained from a logistic regression model with adjustment for age and sex; 95% CI, 95% confidence interval.

### Stratification analysis of the rs2910164 SNP in miR-146a and risk of psoriasis by selected variables

To explore the possible effects of the rs2910164 SNP in miR-146a on psoriasis features, we performed a stratified analysis of that SNP and risk of psoriasis (Table 3). The combined (CG + GG) genotype frequency of miR-146a-rs2910164 was significantly higher among subgroups of male, onset age >40 years, course >12 months, PASI >20 and with family history than among controls (*P* = 0.020; *P* = 0.006; *P* = 0.013; *P* = 0.019; and *P* < 0.001, respectively). When we used the CC genotype as the reference, the increased risk of psoriasis associated with the combined (CG + GG) genotype of miR-146a-rs2910164 was more obvious among subgroups of male (adjusted OR, 1.48; 95% CI, 1.04–2.12), onset age >40 years (adjusted OR, 2.84; 95% CI, 1.17–6.88), course >12 months (adjusted OR, 1.43; 95% CI, 1.06–1.92), PASI > 20 (adjusted OR, 3.01; 95% CI, 1.15–7.88) and with family history (adjusted OR, 2.58; 95% CI, 1.53–4.34).

**Table 3:** Stratified analysis of the rs2910164 genotypes in miR-146a and psoriasis risk by using select variables

Variables	miR-146a (case/control)		Crude OR (95% CI)	Adjusted OR (95% CI)*	*P*†
	CC, *n* (%)	CG + GG, *n* (%)			
Total	132/185 (25.3/31.8)	389/397 (74.7/68.2)	1.37 (1.06–1.79)	1.38 (1.06–1.80)	0.018
Gender					
Female	62/77(47.0/41.6)	174/179 (44.7/45.1)	1.21 (0.81–1.79)	1.23 (0.83–1.83)	0.349
Male	70/108 (53.0/58.4)	215/218 (55.3/54.9)	1.52 (1.07–2.17)	1.48 (1.04–2.12)	0.020
Onset (years)					
≤40	126/185 (95.5/100)	348/397 (89.5/100)	1.29 (0.98–1.68)	1.30 (0.99–1.70)	0.065
>40	6/185 (4.5/100)	41/397 (10.5/100)	3.18 (1.33–7.63)	2.84 (1.17–6.88)	0.006
Course (months)					
≤12	44/185 (33.3/100)	114/397 (29.3/100)	1.21 (0.82–1.78)	1.34 (0.90–2.00)	0.342
>12	88/185 (66.7/100)	275/397 (70.7/100)	1.46 (1.08–1.96)	1.43 (1.06–1.92)	0.013
PASI					
≤20	97/185 (73.5/100)	253/397 (65.0/100)	1.22 (0.91–1.63)	1.22 (0.91–1.63)	0.190
>20	35/185 (26.5/100)	136/397 (35.0/100)	2.98 (1.14–7.77)	3.01 (1.15–7.88)	0.019
Family history					
Yes	19/185 (14.4/100)	106/397 (27.2/100)	2.60 (1.55–4.37)	2.58 (1.53–4.34)	<0.001
No	113/185 (85.6/100)	283/397 (72.8/100)	1.17 (0.89–1.55)	1.18 (0.89–1.56)	0.267

* Odds ratios (ORs) were obtained from a logistic regression model with adjustment for age and sex; 95% CI, 95% confidence interval.

† Two-sided chi-squared test for either genotypes distributions between the cases and controls.

### The rs2910164 SNP can affect the level of mature miR-146a

**Figure 2:**
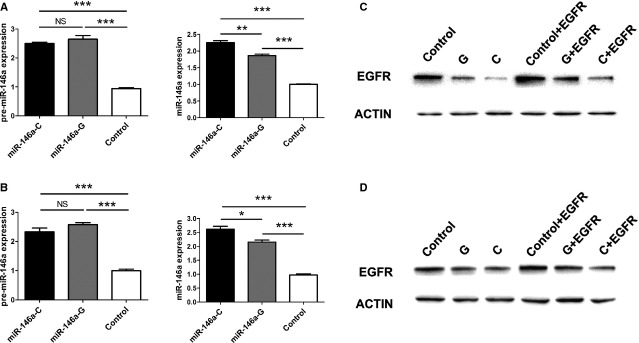
The rs2910164G allele in miR-146a down-regulates the expression of mature miR-146a, and attenuates its inhibitory effect on the expression of *EGFR*. The cells transfected by miR-146a-C plasmids showed significantly higher expression of mature miR-146a, but not pre-miR-146a, than the cells transfected by miR-146a-G plasmids (**A** for HaCaT cells and **B** for normal human keratinocytes). Western blot analysis showed that the rs2910164 miR-146a-C obviously decreased the EGFR protein expression, whereas the inhibitory effect of miR-146a-C is comparatively weaker (**C** for HaCaT cells and **D** for normal human keratinocytes). Transfection with *EGFR* plasmids successfully re-induced the expression of *EGFR* protein. β-actin was used as the internal control. *P < 0.05, **P < 0.01, ***P < 0.001.

It has been demonstrated that SNPs in miRNAs can influence their expression. To clarify whether rs2910164 has that regulatory function, the miR-146a expression plasmids (rs290164G or C allele) were transfected into HaCaT cells and normal human keratinocytes, and the level of pre-miR-146a and mature miR-146a were tested by using qRT-PCR. We found that the cells transfected by both plasmids showed a similarly up-regulated level of pre-miR-146a compared with control (*P* > 0.05). However, the expression of mature miR-146a is significantly higher in the cells transfected by miR-146a-C plasmids compared with that in the cells transfected by miR-146a-G plasmids (Fig. 2A and B).

### The rs2910164G allele in miR-146a impairs its regulation on the expression of *EGFR* in human keratinocytes

Epidermal growth factor receptor is an important gene related to the proliferation of keratinocytes in psoriasis, and it has been validated to be a target gene of miR-146a 23.To evaluate whether the rs2910164G>C change in miR-146a affects its post-transcriptionally regulatory effect on *EGFR* expression, the miR-146a expression plasmids (rs2910164G or C allele) were transfected into HaCaT cells and normal human keratinocytes. As shown in Figure 2C and D, the transfection with the miR-146a expression plasmids led to decreased expression of *EGFR* in cells compared with the control. Moreover, HaCaT cells and normal human keratinocytes transfected by the miR-146a expression plasmids carrying the G allele (the pEX-5-miR-146a-G) showed higher protein level of *EGFR* than that in the cells transfected with the plasmids carrying the C allele (the pEX-5-miR-146a-C).

### The rs2910164G allele in miR-146a weakens its inhibitory effect on the proliferation of human keratinocytes

**Figure 3:**
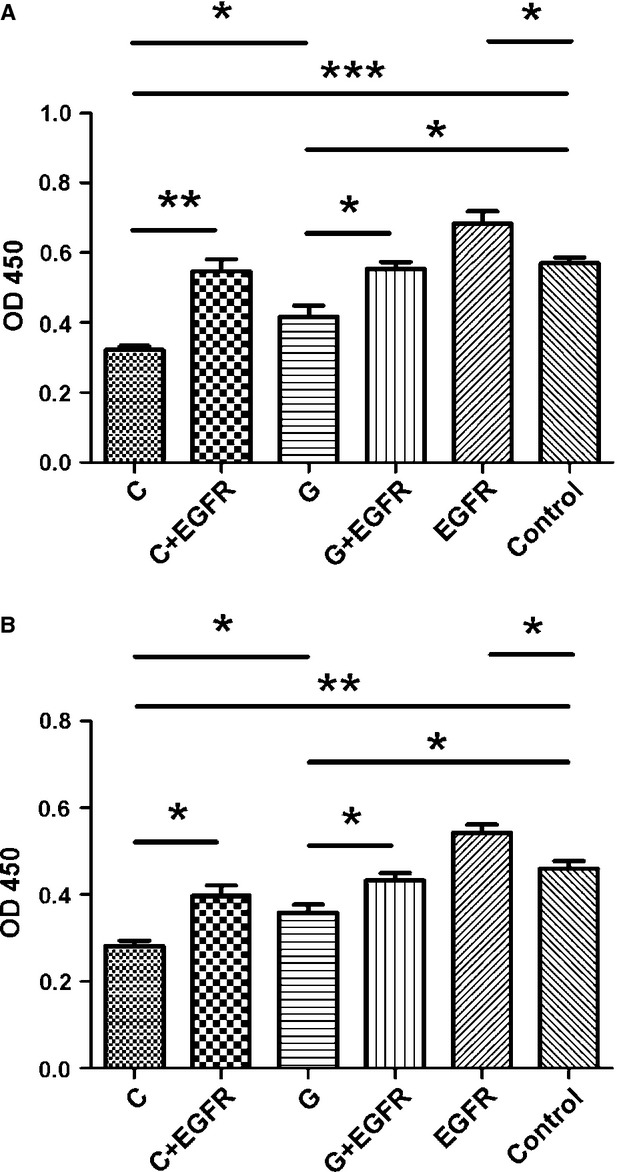
The rs2910164G allele in miR-146a weakens the inhibition on the proliferation of (**A**) HaCaT cells and (**B**) normal human keratinocytes. Re-introduction of *EGFR* was performed with the *EGFR* expression plasmids. The fluorescence intensity of the cells was calculated relative to that of the control. The mean ± SD values of triplicate experiments have been provided. *P < 0.05, **P < 0.01, ***P < 0.001.

To determine whether the rs2910164G>C change in miR-146a affects its regulation on the proliferation of human keratinocytes, the miR-146a expression plasmids (rs2910164G or C allele) were transfected into HaCaT cells and normal human keratinocytes, and CCK8 assays were performed. The proliferation of cells was suppressed after transfection (Fig. 3). However, the miR-146a-G showed a significantly weaker inhibitory effect on the proliferation of both HaCaT cells and normal human keratinocytes than miR-146a-C.

### Re-introduction of *EGFR* rescues the inhibition on the proliferation of keratinocytes induced by miR-146a

To ensure that miR-146a inhibits the proliferation of keratinocytes *via* down-regulating the protein expression of *EGFR*, HaCaT cells and normal human keratinocytes overexpressing miR-146a (C or G) were transfected with *EGFR* plasmids. The results of Western blot showed that the re-introduction of *EGFR* is successful (Fig. 2C and D). Using CCK8 assays, we found that ectopic expression of *EGFR* significantly promoted the proliferation of keratinocytes compared with cells transfected with miR-146a plasmids alone (Fig. 3).

### Association of the miR-146a G/C polymorphism with the mature miR-146a level in psoriatic and normal skin

**Figure 4:**
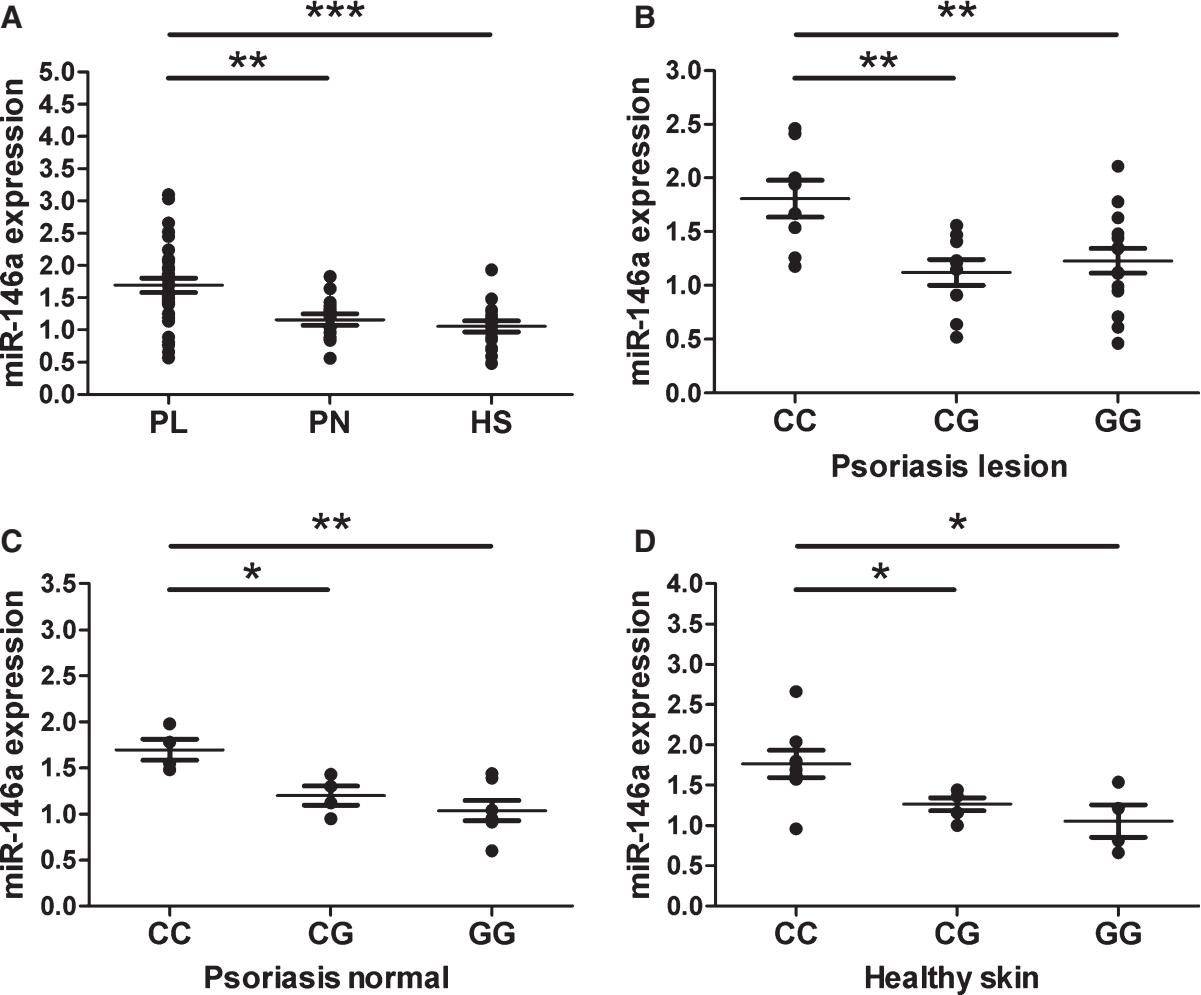
MiR-146a levels in skin samples from psoriasis patients and healthy individuals according to genotypes. (**A**) The expression of miR-146a is significantly higher in psoriatic lesions than in normal skin from psoriasis patients or healthy controls. PL stands for psoriasis lesion, PN stands for psoriasis normal, and HS stands for healthy skin. (**B**) The patients with CG/GG genotypes showed significantly lower miR-146a levels relative to those with CC genotypes. The level of miR-146a in (**C**) normal skin from psoriasis patients and (**D**) Healthy controls shows a similar tendency according to different genotypes. Horizontal line, mean value of each genotype. *P < 0 .05, **P < 0.01, ***P < 0.001.

To test the level of mature miR-146a in psoriatic and normal skin, normal skin specimens were collected from 17 healthy individuals, and psoriatic lesions were collected from 32 psoriasis patients, 15 of whom also agreed to provide their non-lesional skin samples. Resorting to qRT-PCR, we detected significantly increased miR-146a levels in psoriatic lesions compared with healthy skin or non-lesional skin from psoriasis patients. Moreover, the patients with a GG or CG genotype showed significantly lower miR-146a levels in their psoriatic lesions than patients with a CC genotype, which was also observed in non-lesional skin specimens from psoriasis patients and healthy individuals (Fig. 4).

## Discussion

In this hospital-based case–control study, we investigated the association of the rs2910164 SNP in miR-146a with the risk of psoriasis in a Han Chinese population. The data demonstrated that compared with the miR-146a rs2910164 CC genotype, the combined rs2910164 variant genotypes (CG + GG) were associated with an increased risk of psoriasis. We then performed additional experiments to further understand the molecular mechanism underlying this observed association. We found that the rs2910164G allele in miR-146a down-regulates the level of mature miR-146a, impairs its inhibitory regulation on the *EGFR* expression in human keratinocytes, and thus promotes the growth of human keratinocytes. Moreover, skin samples from psoriasis patients and healthy individuals with a GG or CG genotype showed lower expression level of miR-146a than that from individuals with a CC genotype. To the best of our knowledge, this is the first report to demonstrate that the rs2910164 SNP in miR-146a may influence its regulatory function in human keratinocytes and affect the risk of psoriasis in Chinese populations.

Single nucleotide polymorphisms located in the miRNAs are likely to affect the expression of the miRNAs themselves 12,25 and their regulation of target genes 13,16, thus, contribute to the development of various diseases. As a common SNP, the rs2910164 polymorphism in pre-miR-146a has been confirmed to be associated with the risk of various cancers, such as gastric cancer, prostate cancer, thyroid cancer, *etc*. 19,20,23. Intriguingly, recent studies on the association between the SNP and autoimmune diseases (ADs) showed discrepant data. Gazouli *et al*. reported that the miR-146a rs2910164 CC genotype and C allele frequencies were significantly higher in patients of Crohn’s disease than in controls 21. However, in a recent meta-analysis study, Chen *et al*. demonstrated that increased risk of ADs was significantly associated with rs2910164 CG genotype in Asians 22. Consistent with the latter study, we found that individuals carrying the combined (CG + GG) genotypes of rs2910164 in miR-146a had a higher risk of psoriasis, suggesting that the rs2910164G allele in miR-146a may play a role in the aetiology of psoriasis, possibly because the regulatory function of miR-146a is influenced by the rs2910164G allele.

Epidermal growth factor receptor is a gene overexpressed in psoriasis that can promote the proliferation of keratinocytes 6,8, thus contributing to the development of psoriasis. In 2011, Kogo *et al*. reported that miR-146a could regulate the protein expression of *EGFR* by binding to the 3′UTR of *EGFR* messenger RNAs in a study on gastric cancer 23. This observed regulation has been confirmed by an even recent study, which demonstrated that miR-146a could suppress tumour growth and progression by inhibiting protein expression of *EGFR* 26. In accordance with their findings, we also observed the inhibitory regulation of miR-146a on the expression of *EGFR* in keratinocytes in the present study. Moreover, the inhibition was more obvious in cells transfected by miR-146a-C plasmids than miR-146a-G plasmids. Consistent with this observation, we then found that miR-146a-C showed stronger inhibition on growth of keratinocytes than miR-146a-G. In addition, the cells overexpressing miR-146a-C showed significantly higher level of mature miR-146a than the cells overexpressing miR-146a-G. As located in pre-miR-146a, the rs2910164 SNP could not affect the combination between mature miR-146a and *EGFR* mRNA. Therefore, rs2910164G allele in miR-146a may contribute to the increased expression of *EGFR* in psoriasis by down-regulating the level of mature miR-146a, which in turn induces the hyperproliferation of keratinocytes in psoriatic lesions.

Specific miRNA expression profiles have been identified in psoriasis lesions, and some miRNAs (*e.g*., miR-125b, miR-31 and miR-369-3p) have been confirmed to be related with the genesis of psoriasis 27–29. Some high-throughput studies have implicated that miR-146a is increased in psoriatic lesions 30,31, which was also detected in the current study. However, whether miR-146a acts as a disease promoter or a protective factor in psoriasis is not clarified. Xia *et al*. reported that increased miR-146a with impaired function failed to repress the expression of IL-1 receptor-associated kinase 1(*IRAK1*), thus inducing IL-17 persistence in psoriatic skin lesions 32. However, they did not provide experimental evidence supporting that the function of miR-146a is impaired in psoriasis. Indeed, in the present study, we proved that miR-146a can inhibit the expression of *EGFR* and the proliferation of keratinocytes, and the results of protector experiments demonstrate that the suppression of *EGFR* expression is the key mechanism by which miR-146a inhibits the proliferation of keratinocytes. Considering the fact that EGFR is overexpressed in psoriasis, the up-regulation of miR-146a may be a protectively negative feedback factor in psoriasis. In addition, we found that the rs2910164G allele in miR-146a seems to attenuate this protective effect, which partly explains why individuals with the rs2910164G allele in miR-146a are more susceptible to psoriasis as previously described.

Several studies have reported that the variations in miRNA precursors can alter the processing of miRNAs, thus affecting the level of mature miRNAs 18,25,33. In the present study, we found that the rs2910164 SNP in pre-miR-146a can affect the level of mature miR-146a in keratinocytes. As for the level of miR-146a *in vivo*, Kogo *et al*. reported that the expression of mature miR-146a in gastric cancerous tissues from patients carrying a CG/GG genotype is lower than patients carrying a CC genotype 23. Another study on breast cancer also obtained similar results 17. Consistent with their results, we found that the psoriasis patients with the rs2910164G allele in miR-146a had lower lesional expression of mature miR-146a than patients carrying a CC genotype. This phenomenon may be related to the fact that patients carrying the rs2910164 CG/GG genotype have longer disease courses and higher PASI scores as described before. Moreover, we observed the same phenomenon in non-lesional skin from psoriasis patients and healthy individuals, which demonstrates that the regulatory effect of rs2910164 on the level of mature miR-146a is stable. Further investigations are needed to clarify the molecular mechanisms of how this SNP affects the expression of mature miR-146a.

Genetic polymorphisms often vary with ethnicity. In the present study with 582 healthy controls, that frequencies of the hsa-mir-146a GG genotype (16.7%), CG genotype (51.5%), and CC genotype (31.8%) were similar with the frequencies of 19.3%, 53.6% and 27.1%, respectively, observed by Xu *et al*. in 280 Han controls 19, while the reported frequencies in non-Hispanic Caucasian population were 58%, 35.8% and 6.2%, respectively 34. Another recent study on the association between the same SNP and thyroid cancer in Chinese Han population also reported a distribution of the three genotypes similar with our data 20. Such consistence on the genotype frequencies between published studies of other Chinese population and the current study also demonstrates that our results are unlikely to be biased because of selection bias or experimental errors.

In summary, our results demonstrate that the rs2910164 CG/GG genotype in miR-146a was associated with a significantly higher risk of psoriasis than the rs2910164 CC genotype. Moreover, we found that the rs2910164G allele in miR-146a attenuates the inhibitory regulation of miR-146a on the proliferation of keratinocytes probably because its inhibition on the *EGFR* expression is impaired. However, the regulation of increased miR-146a on other target genes in psoriasis remains to be further studied, which can help to clarify the comprehensive role of miR-146a in the pathogenesis of psoriasis and evaluate its possibility to work as a potential therapeutic target in the treatment of psoriasis.
